# Effect of Versenium Hydrogensulfate on Properties of Nickel Coatings

**DOI:** 10.3390/ma16114101

**Published:** 2023-05-31

**Authors:** Marek Baraniak, Grzegorz Lota, Jarosław Wojciechowski, Filip Walkiewicz, Magdalena Regel-Rosocka

**Affiliations:** 1 Institute of Chemistry and Technical Electrochemistry, Faculty of Chemical Technology, Poznan University of Technology, ul. Berdychowo 4, 60-965 Poznan, Poland; marek.baraniak@put.poznan.pl (M.B.); grzegorz.lota@put.poznan.pl (G.L.); jaroslaw.g.wojciechowski@put.poznan.pl (J.W.); 2 Łukasiewicz Research Network, Institute of Non-Ferrous Metals Division in Poznan, Central Laboratory of Batteries and Cells, Forteczna 12, 61-362 Poznan, Poland; 3 Institute of Chemical Technology and Engineering, Faculty of Chemical Technology, Poznan University of Technology, ul. Berdychowo 4, 60-965 Poznan, Poland; filip.walkiewicz@put.poznan.pl

**Keywords:** nickel coatings, versenium cation, EDTA salt additive, corrosion resistance

## Abstract

The salt of formula [H_2_EDTA^2+^][HSO_4_^−^]_2_ (dihydrogen ethylenediaminetetraacetate di(hydrogen sulfate(VI)) was used to examine the physicochemical properties of the resulting Ni layer and evaluate the applicability of the salt as a new additive for Watts-type baths. The Ni coatings deposited from baths containing [H_2_EDTA^2+^][HSO_4_^−^]_2_ were compared with those obtained from other baths. The nucleation of nickel on the electrode was proven to occur the slowest in the bath that contained the mixture of [H_2_EDTA^2+^][HSO_4_^−^]_2_ and saccharin compared with other baths. The addition of [H_2_EDTA^2+^][HSO_4_^−^]_2_ alone (bath III) generated a coating with a morphology similar to that obtained from bath I (without additives). Despite the similar morphology and wettability of the Ni-coated surfaces plated from various baths (all Ni coatings were hydrophilic with contact angles in the range of 68 to 77°), some differences in electrochemical properties were noted. The corrosion resistance for the coatings plated from baths II and IV containing saccharin (I_corr_ = 1.1 and 1.5 µA/cm^2^, respectively) and the mixture of saccharin and [H_2_EDTA^2+^][HSO_4_^−^]_2_ (I_corr_ = 0.88 µA/cm^2^), respectively, was similar or even better than the coating obtained from baths without [H_2_EDTA^2+^][HSO_4_^−^]_2_ (I_corr_ = 9.02 µA/cm^2^).

## 1. Introduction

Nickel coatings are one of the most important and commonly used galvanic coatings. For example, the share of this type of layer in Poland in 2000 amounted to approximately 12.2% of the total surface of details subjected to galvanic deposition [1]. The common characteristics of this type of coating are the result of its physicochemical properties. Nickel coatings are characterized by very good resistance to weather conditions, as well as good mechanical properties, e.g., hardness and aesthetic appearance. They are used as protective (anticorrosive) as well as decorative and technical layers, hardening soft metals, and alloys [2,3,4]. There are several types of technically used acid baths, among which the most popular is the Watts type bath, which is based on nickel(II) sulfate(VI), nickel(II) chloride, and boric acid. Moreover, chloride, fluoroborate, and sulfamic baths are also applied. In the case of each of the above baths, in addition to the basic substances, various additives are used at low concentrations to improve the properties of the coating. Wetting substances, limiting internal stresses, gloss-forming additives, etc., are introduced. They give and increase gloss by increasing the fine crystallinity of the obtained product and the level of microsurface irregularities [2,3,4,5,6,7]. In addition, nickel coatings change the surface properties of coated materials, e.g., stable superhydrophobic coatings with antifouling and anticorrosion functions can be deposited on carbon steel or low-carbon steel [8,9]. Recent investigations have focused on various additives to Ni plating baths to improve the mechanical, tribological, and corrosion performance of such metal coatings compared with that of pure metallic coatings. These additives include graphene oxide (GO), which successfully deposits Ni-GO nanocomposite coating [10,11,12]; various organic compounds, among them acids, salts; or deep eutectic solvents [13,14,15].

Strong complexing agents are added to the plating baths to produce a fine-grained precipitate or modify the microstructure, consequently changing the physicochemical properties of the surface, preventing electroless deposition of metal cations on the cathode (immersion deposition), improving the throwing power of the bath, and increasing the solubility of slightly soluble metal solids. In the case of alloys, complexing agents can affect metal deposition by shifting their potential. The difference in the deposition potential between metals decreases, and it is possible to produce alloy coatings [2,16]. For example, L-proline has been proposed as a complexing agent in a Watts-type nickel bath to improve Ni coating morphology and corrosion resistance [17]. The presence of L-proline indicated a catalytic effect on the reduction of Ni(II), resulting in a great shift in the deposition potential toward more noble potentials. In addition, the surface morphology was affected by the presence of L-proline, and fine, homogeneous, tightly-packed, non-porous grains were obtained on the entire cathode surface. Another additive to a Watts-type bath, i.e., ascorbic acid, significantly improved the microhardness, throwing power, and corrosion resistance of the deposited Ni coatings [18]. Different from L-proline, ascorbic acid addition shifted the polarization curves toward the less noble direction, inhibiting the Ni deposition. Such a phenomenon was assigned to the adsorption of ascorbic acid on the cathode active sites, resulting in the prevention of Ni deposition.

Ethylenediaminetetraacetic acid (EDTA) has been used as a strong complexing agent in various electrolytic baths to improve their stability (avoid precipitation); control the rate of coating deposition; and adjust the crystallization mechanism and the uniformity of the film texture and brighteners [19,20,21,22,23]. However, EDTA is not a common additive in nickel plating baths. For example, information is contained in patents regarding nickel electrodeposition in an acid environment with the addition of EDTA and a powder substance in order to obtain the so-called satin. The use of EDTA as an additive has also been postulated in alkaline baths, which, however, have not been used in practice [6,7]. In addition, the use of EDTA is limited due to its low biodegradability and long residence time in many aqueous systems [24]. In summary, on the one hand, strong complexing agents can and most commonly affect the deposition process and properties of coatings; on the other hand, many are toxic (e.g., CN-) or poorly environmentally friendly (like EDTA). Therefore, ionic liquids (ILs), low-melting organic salts composed of an organic cation, e.g., tetraalkylammonium, 1,3-dialkylimidazolium, and an organic or inorganic anion, have been investigated as new additives to electrolytes due to their almost unlimited number of possible formulations and properties as well as their lower toxicity and greater environmental friendliness than traditional complex compounds [5,25,26,27,28,29,30].

In this trend of searching for new, more environmentally friendly complexing compounds for nickel plating, especially those based on the ionic nature, we proposed [H_2_EDTA^2+^][HSO_4_^−^]_2_ (dihydrogen ethylenediaminetetraacetate di(hydrogen sulfate(VI))) to modify the physicochemical properties of Ni films and potentially obtain alloyed and composite types of coatings. The use of EDTA in the form of a salt can affect the weaker complexation of Ni^2+^ ions, and it can biodegrade more rapidly in the environment. In this paper, we present the effect of the addition of the aforementioned compound and saccharin to the Watts bath on the physicochemical and anticorrosive properties of nickel coatings. The coatings were electrodeposited on the surface of low-carbon steel. The addition of the proposed salt affects the microstructure, electrochemical, and physicochemical properties of the nickel coatings.

## 2. Materials and Methods

The coatings were deposited on AISI 1018 steel. The anodes were nickel sheets of >99% purity. The plating bath was made up of the following chemicals: NiSO_4_∙7H_2_O, NiCl_2_∙6H_2_O, H_3_BO_3_, saccharin, and NaCl, all p.a., purchased in POCH/Avantor (Gliwice, Poland). In order to synthesize the versenium salt, ethylenediaminetetraacetic acid (EDTA, p.a.) (Sigma Aldrich, Poznan, Poland) and 98% H_2_SO_4_ (p.a., POCH/Avantor, Gliwice, Poland) were used.

The versenium salt of the formula [H_2_EDTA^2+^][HSO_4_^−^]_2_ (dihydrogen ethylenediaminetetraacetate di(hydrogen sulfate(VI))) was synthesized by reacting ethylenediaminetetraacetic acid (EDTA) with a 30% solution of sulfuric(VI) acid in a molar ratio of 1:2 according to the following reaction:
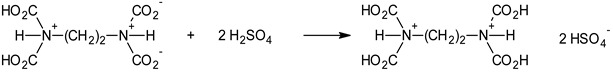
(1)

The reaction was carried out in a suspension for 48 h at room temperature with vigorous stirring. The product, in the form of a white precipitate, was filtered off. After filtration, the product was dried under reduced pressure at 60 °C for 24 h. The yield of the reaction was equal to 95%. The product synthesized was analyzed thermogravimetrically (TGA) using a Mettler Toledo STARe TGA/DSC1 unit (Leicester, UK) under nitrogen.

For the deposition of the nickel coatings, a Watts-type bath was used with the basic composition: 250.1 g/dm^3^ NiSO_4_∙7H_2_O, 31.1 g/dm^3^ NiCl_2_∙6H_2_O and 40.8 g/dm^3^ H_3_BO_3_. The electrolyte was prefiltered. The basic bath (marked bath I) did not contain additives, while baths II-IV contained additives of saccharin (15 mM), [H_2_EDTA^2+^][HSO_4_^−^]_2_ (6 mM), and mixed saccharin (15 mM) and [H_2_EDTA^2+^][HSO_4_^−^]_2_ (6 mM), respectively. The addition of saccharin was proposed on the basis of previous research [4]. The baths were thermostated at 323 K (50 °C) (Huber CC-K6 thermostat/cryostat, Raleigh, NC, USA).

A standard Hull cell (cell volume 0.25 dm^3^) was used for preliminary tests to select the Ni deposition parameters. A current of 2 A was applied. Coatings with good electrical performance were obtained in the range of cathodic current densities of 2 to 10 A/dm^2^. The 12 cm^2^ elements were cut out of steel and subjected to mechanical cleaning with 2500 grit sandpaper, chemical degreasing in acetone, anodic electrochemical degreasing in a solution containing 15 g/dm^3^ NaOH and 30 g/dm^3^ Na_3_PO_4_∙H_2_O (298 K, 10 min), and rinsing with deionized water. Then, after chemical etching in a 1 M HCl solution (298 K, 5 min) and rinsing twice with deionized water, the elements were placed in a nickel plating bath without drying.

On the basis of the parameters selected in the Hull cell experiments, the coating deposition process was carried out in a vessel with a capacity of approximately 0.5 dm^3^ using a laboratory DC power supply DC 3010D-II 2x30V/10A (MCP lab electronics, Shanghai, China). The cathodic current density I_k_ and the deposition time were equal to 10 A/dm^2^ and 25 min, respectively. The bath was not stirred.

The coated samples (at least three from each bath) were analyzed using optical microscopy (Delta Optical MET-200-TRF metallographic microscope, Delta Optical, Poland), scanning electron microscopy (SEM), energy-dispersive spectroscopy (EDAX) (Quanta 250 FEG, an FEI ThermoFisher scientific device with Energy Dispersive Spectroscopy module, Hillsboro, OR, USA), water contact angle tests using the deposited drop method (DropMaster DM 100 device with a digital camera, Kyowa, Japan), and corrosion tests.

Three-electrode DC and AC corrosion tests were carried out in a 3.5% NaCl aqueous solution (0.15 dm^3^) at ambient temperature without stirring using the VMP-III BioLogic potentiostat/galvanostat (BioLogic, Seyssinet-Pariset, France) with EC-Lab v. 11.10 software. The area of the working electrode was equal to 0.4 cm^2^. A saturated calomel electrode (SCE) and platinum were used as the reference and auxiliary electrodes, respectively. The corrosion tests, which included open circuit potential measurement (OCP) for 4 h, a linear sweep voltammetry (LSV) method in the range of −100 to 500 mV vs. OCP (scan rate of potential change 0.2 mV/s), corrosimetry (CM) (range of potential change ±25 mV vs. OCV), and electrochemical impedance spectroscopy (EIS) (BioLogic, Seyssinet-Pariset, France) (frequency range 200 kHz–20 mHz, amplitude ±10 mV), were carried out twice.

On the basis of electrochemical measurements, the values of corrosion potentials (E_corr_), corrosion current densities (j_corr_), polarization slopes (β_a_ and β_c_), polarization resistance (R_p_), and charge transfer resistance (R_ct_) were determined. In the case of voltamperometry, the method of extrapolation of Tafel curves was used (E_corr_, j_corr_, β_a_, and β_c_). Although corrosimetry uses a much narrower range of polarization, it allows the values of j_corr_, R_p_, β_a_, and β_c_ to be calculated. The relationship between these parameters is expressed by the Stern–Geary equation, which is also used by EC-Lab Software v. 11.43^®^. Nyquist plots were obtained by means of impedance spectroscopy and theoretical data fitting using EC-Lab Software^®^. This action results in the selection of electric equivalent circuits, which in turn contain elements that can be used to describe electrochemical phenomena.

## 3. Results

### 3.1. Characterization of the Synthesized Versenium Salt

**Figure 1:**
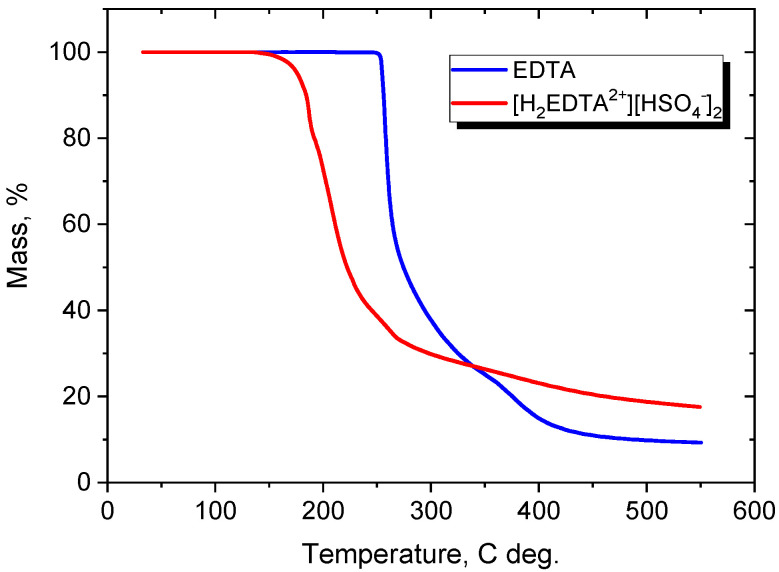
Comparison of the thermal decomposition curves of (**------**) pure EDTA and (**------**) the [H_2_EDTA^2+^][HSO_4_^–^]_2_ salt.

The synthesized [H_2_EDTA^2+^][HSO_4_^−^]_2_ was a white amorphic solid. The thermogravimetric analysis was carried out to determine the thermal stability of the obtained product. The results of the TGA analysis (Figure 1) showed significant differences between pure EDTA and the [H_2_EDTA^2+^][HSO_4_^−^]_2_ salt.

For example, the thermal stability for pure EDTA, defined as a 1% weight loss (T0.01), is 253 °C, whereas the introduction of two H_2_SO_4_ molecules and the transformation of EDTA into [H_2_EDTA^2+^][HSO_4_^−^]_2_ caused a reduction in this temperature to 157 °C. For EDTA, two steps of degradation were observed. The first step was characterized by a sharp decrease in the temperature range of 240–345 °C with a weight loss of 74%, while the second step occurred in the range of 345–550 °C with a mass loss of another 17%. For [H_2_EDTA^2+^][HSO_4_^−^]_2_, the first step of decomposition occurred at a lower temperature of 150–194 °C (mass loss 19%); the second step, with a mass loss of 38%, was registered at 194–247 °C. Two more decomposition steps occurred at 247–278 °C and 304–540 °C, with mass losses of 7 and 11%, respectively. In contrast to the metal complex systems of EDTA [31] for both cases, pure EDTA and versenium hydrogen sulfate, the endothermic effect of decomposition was observed at around 253 and 180 °C. These mass loss/degradation steps could be attributed to a decomposition and evaporation process rather than a catalytic decomposition reaction as in metal complex systems.

### 3.2. Nickel Plating from Modified Baths

**Figure 2:**
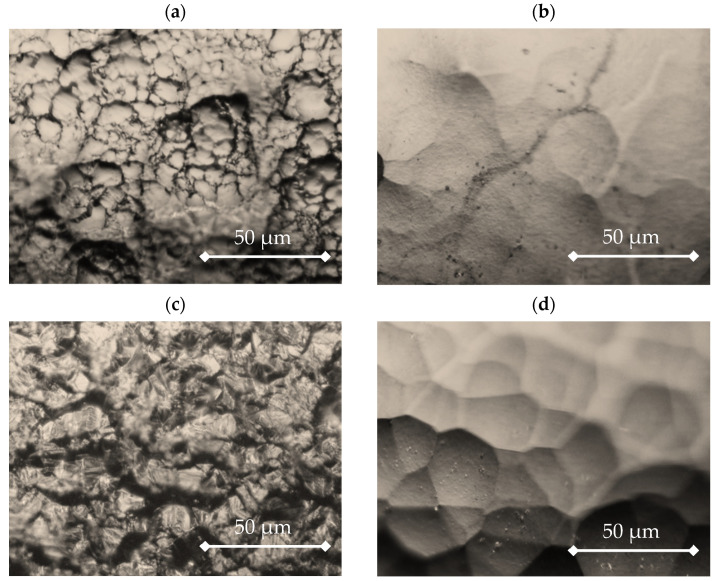
Surface morphology of the nickel coatings obtained from (**a**) bath I (without additives), (**b**) bath II (15 mM of saccharin), (**c**) bath III (6 mM of [H_2_EDTA^2+^][HSO_4_^−^]_2_), and (**d**) bath IV (15 mM of saccharin and 6 mM of [H_2_EDTA^2+^][HSO_4_^–^]_2_). Images were taken by optical microscope.

**Figure 3:**
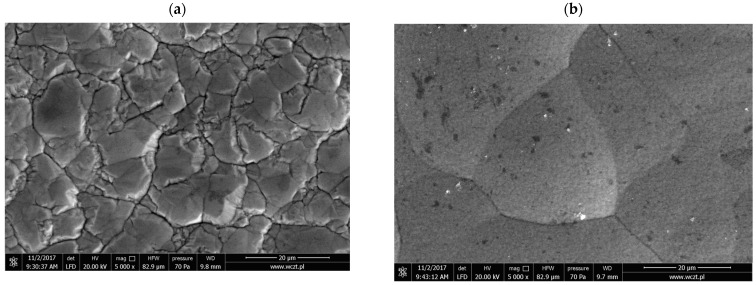

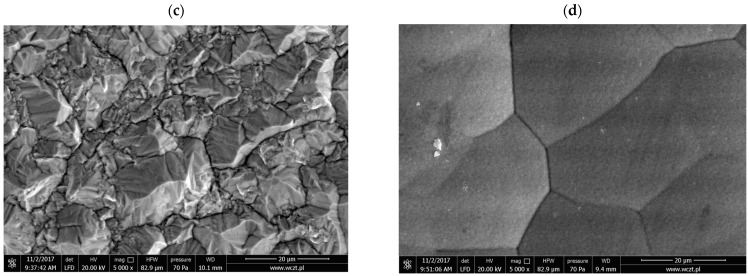
Surface morphology of the nickel coatings obtained from (**a**) bath I (without additives), (**b**) bath II (15 mM of saccharin), (**c**) bath III (6 mM of [H_2_EDTA^2+^][HSO_4_^−^]_2_), and (**d**) bath IV (15 mM of saccharin and 6 mM of [H_2_EDTA^2+^][HSO_4_^−^]_2_). Images were taken by scanning electron microscope.

The steel elements were coated with nickel from various baths, marked as I–IV (for composition, see the Materials and Methods section). Figure 2 and Figure 3 show the surface microstructure of the coatings obtained from the various applied baths using optical microscopy and SEM, respectively.

The coatings obtained from baths II–IV containing additives were increasingly coarse-crystalline, especially in the case of bath IV. In the process of electrocrystallization, two stages of metal layer construction could be distinguished, i.e., the formation of crystallization centers (nucleation) and crystal growth. Both stages had a certain rate depending on the type of metal being deposited and the conditions of electrolysis. The relative rates of formation of crystallization centers and crystal growth determine the properties and appearance of the deposited coating [32]. It was shown by Zhou et al. [33] that during Ni-Co deposition more fine nuclei on the copper surface can be related to its high nucleation rate and a greater number of active nucleation sites. Thus, the images presented in Figure 2 and Figure 3 show that the nickel nucleation on the steel electrode was the slowest in bath IV, which contained the addition of both [H_2_EDTA^2+^][HSO_4_^−^]_2_ and saccharin.

A significant effect of saccharin addition on the surface microstructure was also noted. The addition of [H_2_EDTA^2+^][HSO_4_^−^]_2_ alone (bath III) generated a coating with a morphology similar to that obtained from bath I (without additives), i.e., finer grains than in the presence of saccharin. In addition, other additives to Watts baths, such as ascorbic acid, were shown to modify the texture of the formed Ni coatings and produce more fine grains [18].

By contrast, the coatings obtained from baths II and IV with saccharin addition were similar to each other. The coatings obtained from baths II–IV, containing additives, were increasingly coarse-crystalline, especially in the case of bath IV. This showed that the nickel nucleation on the steel electrode was the slowest in the bath, which contained the addition of both [H_2_EDTA^2+^][HSO_4_^−^]_2_ and saccharin. A significant effect of saccharin addition on the surface microstructure was also noted. The addition of [H_2_EDTA^2+^][HSO_4_^−^]_2_ alone (bath III) generated a coating with a morphology similar to that obtained from bath I (without additives). By contrast, the coatings obtained from baths II and IV with saccharin addition were similar to each other.

**Figure 4:**
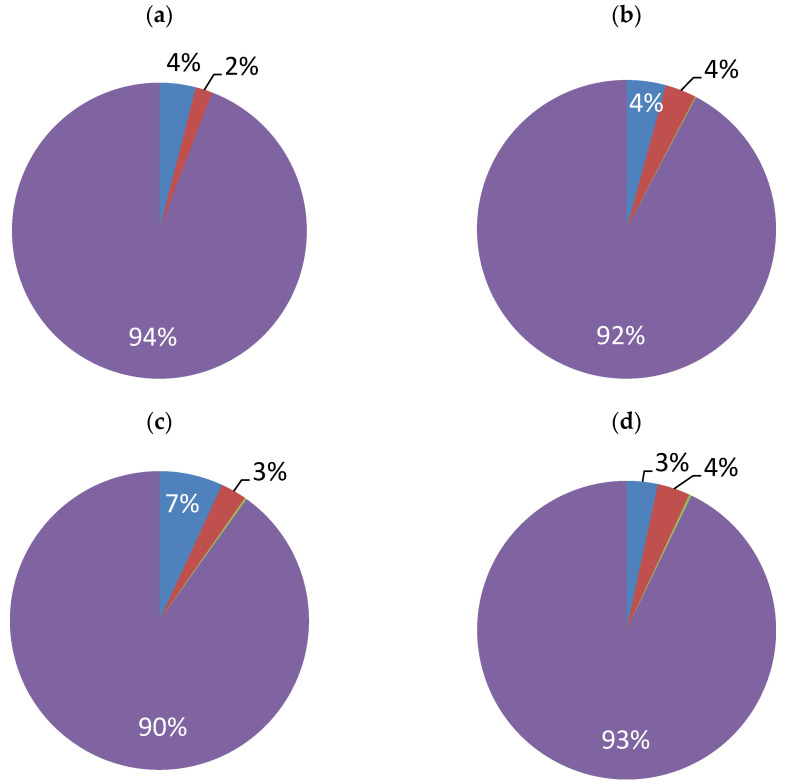
Atomic composition (%) (■—C, ■—O, ■—Ni) of the coatings obtained from (**a**) bath I (without additives), (**b**) bath II (15 mM of saccharin), (**c**) bath III (6 mM of [H_2_EDTA^2+^][HSO_4_^−^]_2_), and (**d**) bath IV (15 mM of saccharin and 6 mM of [H_2_EDTA^2+^][HSO_4_^−^]_2_).

Measurements of the water contact angle (Θ) showed that the coatings obtained from baths with a single additive (baths II or III) had very similar surface characteristics, i.e., they reached average values of Θ equal to 76–77°. However, the coating deposited from bath IV was a little bit less hydrophobic, as the Θ value amounted to 71°. The lowest contact angle (68°) was obtained for the coating deposited from bath I, i.e., without additives. Values of contact angles less than 90° indicate that all Ni-coated surfaces are hydrophilic [34]. However, a slight increase in the wettability of the surface plated from additive-containing baths showed that it was affected not only by the hydrophobic nature of the additive molecules that were co-deposited on the surface but also by the change in the 3D microstructure of the surface (e.g., coarse crystallinity, surface roughness). Slight changes in the composition of the coating (the percentage changes in the content of Ni, Fe, C, and O atoms) were observed by means of the EDS technique (Figure 4).

In all cases, the Fe content in the coating was negligible (<0.25%) (Figure 4). It was also seen that the relative atomic compositions of layers from baths containing saccharin (II and IV) were very similar. The nickel content in the coatings ranged from 90% for bath III to more than 94% for bath I.

The data concerning the presence of carbon were interesting; carbon was the most abundant in the deposited coating apart from nickel. Surprisingly, the largest share of the surface area showed the sample obtained from the solution with the addition of EDTA salt (Figure 4c) and not, as expected, from bath IV, containing both compounds, i.e., saccharin and EDTA salt. The second compound contained more carbon atoms in the molecule, but its concentration was more than two times as low as the concentration of saccharin. Therefore, the share of carbon in the coating formed was determined by the steric and inductive effects of the compounds used. A significant issue is the fact that the EDTA salt contains the EDTA cation, and its movement toward the steel cathode is forced by the strength of the electric field during the cathodic polarization of the steel. Hence, the content of carbon from the organic compound was higher.

**Figure 5:**
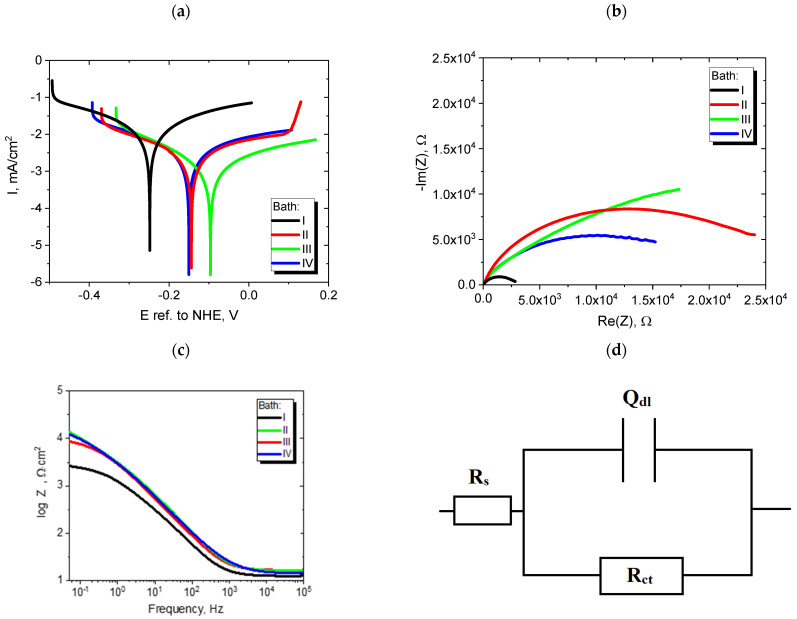
(**a**) Tafel curves, (**b**) impedance spectra, (**c**) Bode curves of Ni coatings plated from baths I–IV, and (**d**) electrical equivalent circuit.

The results of all the electrochemical measurements were consistent with the aforementioned physicochemical analysis (water contact angle and EDAX). Therefore, large differences were observed in the electrochemical properties of the materials plated with Ni from various baths (I–IV). Figure 5a shows the voltammetric curves in the semilogarithmic system, on the basis of which the Tafel curves were determined. Figure 5b–d depict the results (Nyquist and Bode plots) of the electrochemical impedance spectroscopy measurements fitted on the basis of the electrical equivalent circuit. Table 1 summarizes the corrosion parameters obtained using various electrochemical techniques.

A comparison of the values of the corrosion parameters shown in Table 1, obtained by different electrochemical techniques, showed a large convergence of the data obtained for individual samples. Of course, it is not possible to directly compare all the values of the parameters obtained by different techniques. The convergence refers to values obtained in every technique separately. Electrochemical tests indicated that the coatings deposited from baths II–IV were characterized by a significant decrease in the rate of the corrosion process compared with the coating obtained from the unmodified Watts bath I. The lowest value of the corrosion current density was observed for the nickel-coated specimen from bath II containing [H_2_EDTA^2+^][HSO_4_^−^]_2_.

It is also worth noting that this sample contained the largest content of carbon atoms (Figure 4c). The corrosion parameters for the coatings plated from baths II and IV containing saccharin and the mixture of saccharin and [H_2_EDTA^2+^][HSO_4_^−^]_2_, respectively, were similar. In view of similar surface morphology and microstructure, as well as the elemental composition of the coating, this would be expected. However, the corrosion resistance of the surface plated from bath IV was slightly lower because this surface was characterized by a lower hydrophobicity compared with the coating from bath II. An increase in the hydrophobicity of the surface of various metals (for example, Cu and Ni) was shown to improve the corrosion resistance of these materials as a result of reduced wettability with water [9,34,35,36]. Thus, it could be expected that Ni-coated elements from bath IV would be more susceptible to corrosion than the coatings plated from bath II.

The results of the impedance tests also confirmed the above theses. Nyquist curves (Figure 5b) of all samples indicated the presence of a one-time constant that described the formation of an electrical double layer at the electrode/electrolyte interface. Therefore, an equivalent electric circuit was selected (Figure 5d). The elements of this circuit were a resistor R and a parameter Q. The latter describes the impedance of the constant-phase element, which is called an imperfect capacitor, i.e., it depicts a capacitance of, e.g., the electrical double layer at the electrode/electrolyte interface:

(2) $$Z(j\omega )=\frac{1}{{Q(j\omega )}^{\alpha }}$$

where j, ω, and α are the imaginary number, angular frequency, and factor reflecting capacitive dispersion, respectively. The values of the Q_dl_ parameter and the charge transfer resistance R_ct_ at the interface depend on the anticorrosive properties of the nickel coating. A larger diameter of the semicircle in the Nyquist plot graph indicated a higher R_ct_ value and a lower value of non-ideal capacitance (Q_dl_) of samples modified with the addition of EDTA and saccharin (Table 1). In addition, it is worth noting that the value of the impedance modulus on the Bode plots for the lowest frequency value was the lowest for the reference sample (bath I), which proves that its anticorrosive properties were the worst.

## 4. Conclusions

The use of the synthesized acidic salt [H_2_EDTA^2+^][HSO_4_^−^]_2_ as an additive to the Ni plating bath affected the physicochemical and corrosion properties as well as the surface morphology of the deposited layer, despite the low additive concentration. Significantly lower corrosion currents were obtained for the coating prepared from the bath with the addition of only acidic salt [H_2_EDTA^2+^][HSO_4_^−^]_2_ in relation to the pure Watts-type bath and to both electrolytes with saccharin. The coating containing [H_2_EDTA^2+^][HSO_4_^−^]_2_ particles showed the best corrosion resistance, which can be explained by a more fine-grained structure and a higher content of carbon atoms. The structure of EDTA salt and especially the presence of EDTA cation affected the improved anticorrosive properties of the coating. The properties of deposits obtained from both saccharin-containing baths were very similar in terms of morphology as well as electrochemical properties. This proves the definite influence of saccharin in the system on the coating properties. The use of organic salt-containing cations in small concentrations may be an interesting direction for the development of new types of plating baths and modifications of the existing ones.

## Figures and Tables

**Table 1:** Corrosion parameters determined from all the electrochemical techniques for the Ni coatings plated from baths I–IV.

Techniques/Parameter		Ni Coating from			
		Bath I	Bath II	Bath III	Bath IV
Open circuit voltage					
E ref. to NHE	(mV)	−183	−87	−57	−84
Voltamperometry					
E_cor_ ref. to NHE	(mV)	−250	−144	−100	−150
I_cor_	(µA/cm^2^)	12.89	2.70	1.35	2.94
|β_a_|	(mV/dec)	304	370	324	371
|β_c_|	(mV/dec)	283	272	201	271
Corrosimetry					
E_cor_ ref. to NHE	(mV)	−244.0	−89.5	−78.6	−134.5
I_cor_	(µA/cm^2^)	9.02	1.10	0.88	1.50
R_p_	(Ω∙cm^2^)	2891	23,721	29,600	17,402
Electrochemical impedance spectroscopy					
R_ct_	(Ω∙cm^2^)	2997	27,299	42,193	19,454
Q_dl_∙10^−6^	(F∙s^(α−1)^)	69.6	33.9	43.1	36.1

## Data Availability

The data are available upon request.
